# A small-angle X-ray scattering study of alpha-synuclein from human red blood cells

**DOI:** 10.1038/srep30473

**Published:** 2016-07-29

**Authors:** Katsuya Araki, Naoto Yagi, Rie Nakatani, Hiroshi Sekiguchi, Masatomo So, Hisashi Yagi, Noboru Ohta, Yoshitaka Nagai, Yuji Goto, Hideki Mochizuki

**Affiliations:** 1 Department of Neurology, Osaka University Graduate School of Medicine, 2-2 Yamadaoka, Suita, Osaka 565-0871, Japan; 2 Japan Synchrotron Radiation Research Institute (JASRI), SPring-8, 1-1-1 Kouto, Sayo, Sayo, Hyogo 679-5198, Japan; 3 Institute for Protein Research, Osaka University, 3-2 Yamadaoka, Suita, Osaka 565-0871, Japan; 4 Center for Research on Green Sustainable Chemistry, Tottori University, 4-101 Koyamacho-minami, Tottori, Tottori 680-8550, Japan; 5 Department of Neurotherapeutics, Osaka University Graduate School of Medicine, 2-2 Yamadaoka, Suita, Osaka 565-0871, Japan

## Abstract

α-synuclein (α-syn) is the main component of Lewy bodies, which are neuropathological hallmarks of patients with Parkinson’s disease. As it has been controversial whether human α-syn from erythrocytes exists as a tetramer under physiological conditions, we tried solving this issue by the small-angle X-ray solution scattering method. Under two different conditions (high ionic strength with a Tris buffer and low ionic strength with an ammonium acetate buffer), no evidence was found for the presence of tetramer. When comparing erythrocyte and recombinant α-syn molecules, we found no significant difference of the molecular weight and the secondary structure although the buffer conditions strongly affect the radius of gyration of the protein. The results indicate that, even though a stable tetramer may not be formed, conformation of α-syn depends much on its environment, which may be the reason for its tendency to aggregate in cells.

α-synuclein (α-syn) is the main component of Lewy bodies, which are neuropathological hallmarks of patients with Parkinson’s disease and dementia with Lewy body^1^,^2^. In Lewy bodies, α-syn has a fibril-like structure^3^,^4^. α-syn is highly expressed in central nervous system^5^,^6^,^7^ and erythrocyte^8^, but its function is obscure. Many structural analyses, such as circular dichroism, Fourier transform infrared spectroscopy, small angle X-ray scattering (SAXS), revealed that α-syn exists as an intrinsically disordered monomer of about 14.46 kDa^9^,^10^,^11^. For these measurements, recombinant human α-syn purified from *Escherichia coli* (*E. coli*) which has a non-acetylated N-terminal has been used. In 2011, Bartels *et al.* reported that α-syn purified from human red blood cells (RBCs) and cultured cells exists as a stable tetramer that resists aggregation^12^. To study the state of endogenous α-syn in living human cells, they used α-syn prepared from RBCs. Then, Wang *et al.* also reported that α-syn purified from *E. coli* in non-denaturing conditions is a dynamic tetramer^13^. Furthermore, Trexler *et al.* showed that N-terminal acetylation is critical for formation of α-helical oligomers of α-syn^14^. Some papers reported that N-terminal modification or its interaction with lipid menbranes, may change the structure and aggregation of α-syn^15^,^16^. In contrast, in 2012, Fauvet, *et al.* reported that α-syn in central nervous system, erythrocytes, and mammalian cells exists predominantly as a disordered monomer^17^. They also reported that N-terminal acetylation of α-syn does not dramatically affect its structure or oligomerization state *in vitro* and in intact cells^18^. Some of these experiments were performed under different buffer conditions. Although the presence of tetramer may be arguable^19^, the state of non-recombinant α-syn, particularly from RBCs, in solution has not been characterized.

In this study, we examined the oligomeric state of α-syn from human RBCs with SAXS and compared it to recombinant human α-syn expressed in *E. coli* under different buffer conditions.

## Results

### Characterization of the proteins

**Figure 1:**
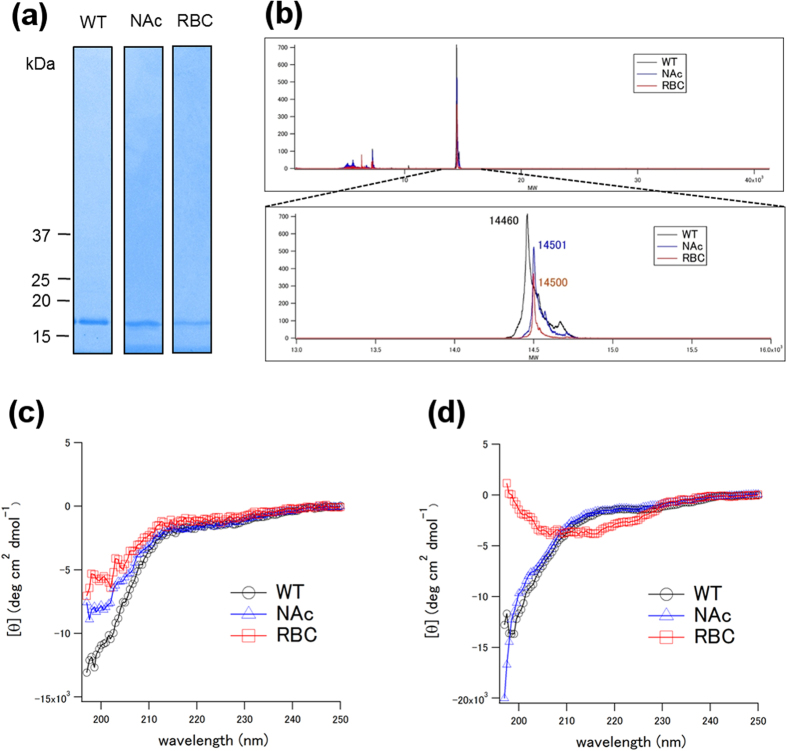
(**a**) SDS-PAGE/Coomassie Brilliant Blue (or silver) staining. WT, NAc and RBC represents wildtype α-syn, N-terminally acetylated α-syn and α-syn purified from human RBCs, respectively. (**b**) MALDI spectra of α-syn. Theoretical molecular weights (MWs) of Wild type (WT) and N-terminally acetylated (NAc) are 14460 and 14502, respectively. RBC represents α-syn purified from human RBCs. (**c**) Circular dichroism spectra of WT, NAc and RBC in the AA buffer (10 mM ammonium acetate (pH 7.4)). (**d**) Circular dichroism spectra of WT, NAc and RBC in the Tris buffer (50 mM Tris-HCl, 150 mM NaCl (pH 7.4))

The purity of the proteins was confirmed by SDS-PAGE and MALDI mass spectroscopy (Fig. 1a,b). They confirmed that the proteins were of high enough purity and that α-syn from human RBC (RBC) was likely to be N-terminally acetylated in terms of molecular weight^12^.

Circular dichroism (CD) spectra of the proteins in the AA buffer (10 mM Ammonium acetate (pH 7.4)) and the Tris buffer (50 mM Tris-HCl (pH 7.4), 150 mM NaCl) are shown in Fig. 1c,d, respectively. The spectra of recombinant wild type α-syn (WT α-syn) showed typical random coil profiles in both buffers. Similarly, those of N-terminally acetylated recombinant α-syn (NAc α-syn) also showed typical random coil profiles in both buffers. On the other hand, while the spectrum of α-syn purified from human RBC (RBC α-syn) showed a typical random coil profile in the AA buffer (Fig. 1c), it suggested the presence of secondary structure, although difficult to analyze in detail, in the Tris buffer (Fig. 1d).

### Static SAXS measurements

**Figure 2:**
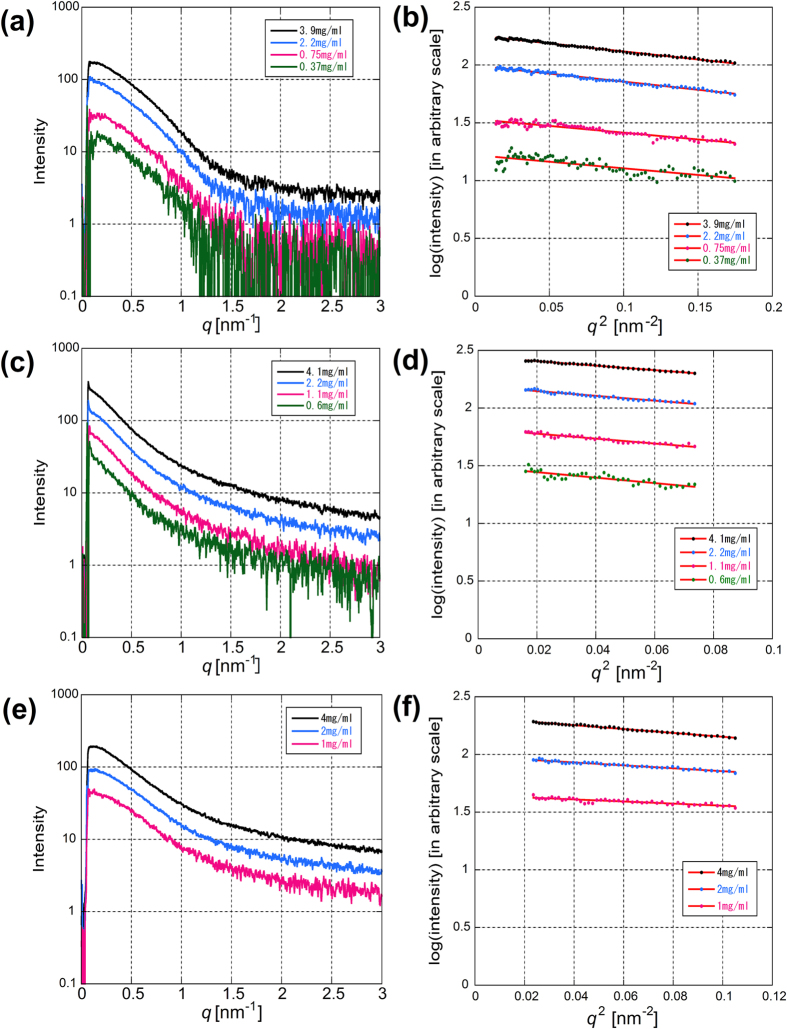
(**a**) A typical set of SAXS profiles from human RBC α-syn at four different concentrations. (**b**) Guinier plots of the data in (**a**). (**c**) A typical set of SAXS profiles from WT α-syn in the Tris buffer at four different concentrations. (**d**) Guinier plots of the data in (**d**). (**e**) A typical set of SAXS profiles from WT α-syn in the AA buffer at three different concentrations. (**f**) Guinier plots of the data in (**e**).

**Figure 3:**
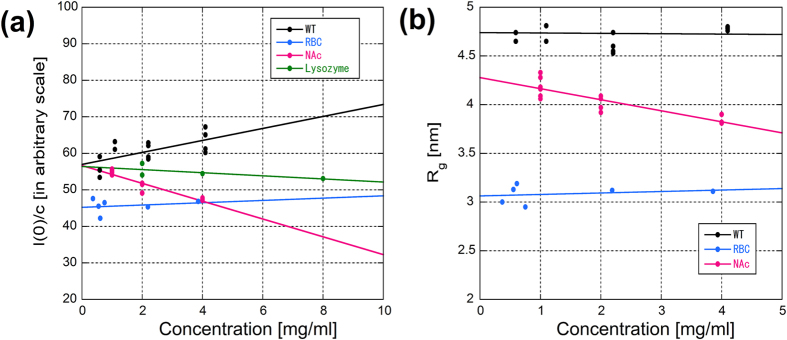
(**a**) Concentration dependence of the R_g_ values in three types of α-syn. The value at the abscissa was used as the R_g_ value at infinite dilution. (**b**) Concentration dependence of the I(0)/c value.

Typical X-ray scattering intensity curves at four different concentrations of RBC α-syn in the Tris buffer are shown in Fig. 2a. The Guinier plot and the linear fitting is shown in Fig. 2b. The concentration dependence of the radius of gyration (R_g_) is plotted in Fig. 3a. When extrapolated to zero concentration, the R_g_ value was 3.31 ± 0.30 nm (mean ± standard deviation, n = 4). In the AA buffer, which was used by Bartels *et al.*^12^, R_g_ was 3.33 ± 0.10 nm (n = 2). Although difference in the CD spectrum (Fig. 1c,d) suggests conformational difference in the two buffers, the R_g_ values were similar, showing that formation of secondary structure does not affect the flexibility or extensibility of the protein.

The I(0)/c (c is protein concentration) values normalized to that of lysozyme, which has a molecular weight (14.3 kDa) similar to α-syn, were 1.14 ± 0.40 (n = 4) in the Tris buffer and 0.94 ± 0.03 (n = 2) in the AA buffer, showing that the protein is monomeric in both buffers.

Typical X-ray scattering intensity curves of recombinant wild type (WT) α-syn in the Tris buffer are shown in Fig. 2c with a Guinier fit in Fig. 2d. From extrapolation to the zero concentration (Fig. 3a), the R_g_ value was obtained as 4.27 ± 0.37 nm (n = 7). Compared with RBC α-syn, the region where a linear fit was available in the Guinier plot was narrower. Typical X-ray scattering intensity curves of recombinant WT α-syn in the AA buffer are shown in Fig. 2e with a Guinier fit in Fig. 2f. In the AA buffer, R_g_ was found to be smaller, 2.72 ± 0.44 nm (n = 3). The I(0)/c values normalized by that of lysozyme were 1.58 ± 0.53 (n = 7) in the Tris buffer and 1.06 ± 0.07 (n = 3) in the AA buffer.

Since α-syn prepared from RBCs has acetylated N-terminus (Fig. 1), N-terminus of recombinant α-syn was acetylated to find if the difference in the SAXS results between RBC and recombinant α-syn is due to this modification. The R_g_ value of N-terminally acetylated recombinant α-syn (NAc) was 4.04 ± 0.22 nm (n = 4) and 3.09 ± 0.18 nm (n = 2) in the Tris and AA buffers, respectively. The I(0)/c values were 1.34 ± 0.15 and 0.99 ± 0.19 in the Tris and AA buffers, respectively. These results are summarized in Table 1.

### SEC-SAXS

**Figure 4:**
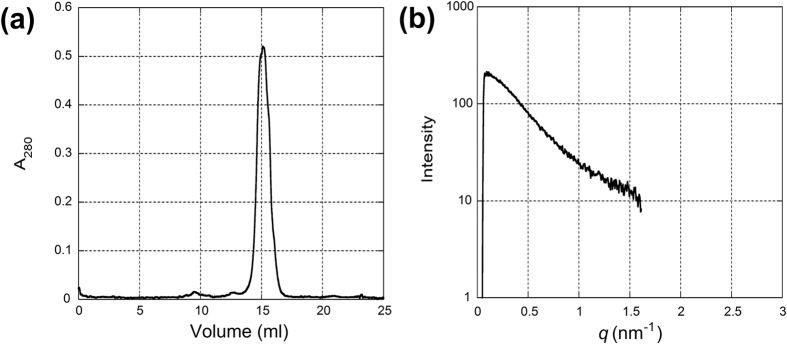
(**a**) An elusion profile of recombinant α-syn from a size-exclusion column. The flow rate was changed from 0.5 to 0.2 ml/min at 12.7 ml. (**b**) An X-ray scattering profile of α-syn pooled around the elusion peak. The data in 30 frames (5 min) across the elusion peak, corresponding to 1.0 ml of elusion volume, were summed for the analysis. The linear region of the Guinier plot was q = 0.145 to 0.359 nm^−1^. The data were truncated at around q = 1.5 nm^−1^ because of the small size of the X-ray detector but plotted so as to be compared with Fig. 1c.

In the static SAXS experiment, small-angle scattering curves from recombinant WT α-syn in the Tris buffer had only a limited region that can be used for the Guinier analysis (Fig. 2c,d). The intensity in the very small angle region (q < 0.2 nm^−1^) was steeper than that from RBC α-syn, suggesting the presence of oligomers or aggregates. To remove these large particles, measurements were made with proteins after passing through a size-exclusion column. Since the amount of protein that can be prepared from RBCs is small, the measurement was made only with recombinant α-syn. The SAXS measurement was made continuously during protein elution and a profile was obtained by summing data acquired when the protein concentration was more than half of its peak (Fig. 4a). The R_g_ value was 3.59 ± 0.14 nm (5 separate experiments). The scattering curve (Fig. 4b) was more similar to that of RBC α-syn obtained in the static experiments than that of recombinant proteins (Table 1). With N-terminally acetylated recombinant α-syn, R_g_ was found to be 3.59 ± 0.28 nm (n = 2).

## Discussion

In this study, we prepared α-syn from RBCs following the procedure by Fauvet *et al.*^17^. The SAXS measurements were made in the buffer condition (the low ionic-strength AA buffer) in which they found tetramers. At the concentration commonly used for the SAXS measurements (a few mg/ml), we found that the protein was monomeric. With both the Tris and the AA buffers and with RBC and recombinant α-syn preparations, no evidence was obtained that suggested the presence of tetramers. These results are in accordance with those of Fauvet *et al.*^17^ who found that RBC α-syn is monomeric. It also agrees with a more recent report that α-syn predominantly exists as a monomer in a living cell^20^.

The two α-syn preparations used in this study, one from RBCs and the N-terminally acetylated one cloned and expressed in *E. coli*, share the same amino acid sequence of human α-syn and N-terminus acetylation. However, the physical characteristics of these preparations were clearly different. RBC α-syn had a smaller R_g_ than the recombinant N-terminally acetylated α-syn in the Tris buffer (Table 1). This may be due to a rather harsh preparation method of the recombinant protein which involves heating up to 90 °C. In fact, it has been shown that recombinant α-syn (with extra 10 residues at the N-terminus) purified without heat denaturation was found more disordered with CD and NMR (Wang *et al.*)^13^. Heat treatment may cause irreversible effects on the α-syn structure.

The X-ray scattering curves of the recombinant α-syn, both WT and N-terminally acetylated, had a narrower linear region compared with the RBC α-syn (Fig. 2c), suggesting it may be a mixture of different conformations or oligomers. Indeed, with a size-exclusion chromatography, the scattering curve became more like one from a monodisperse preparation and the R_g_ value was smaller. The preparation method may have caused partial aggregation or oligomerization.

**Figure 5:**
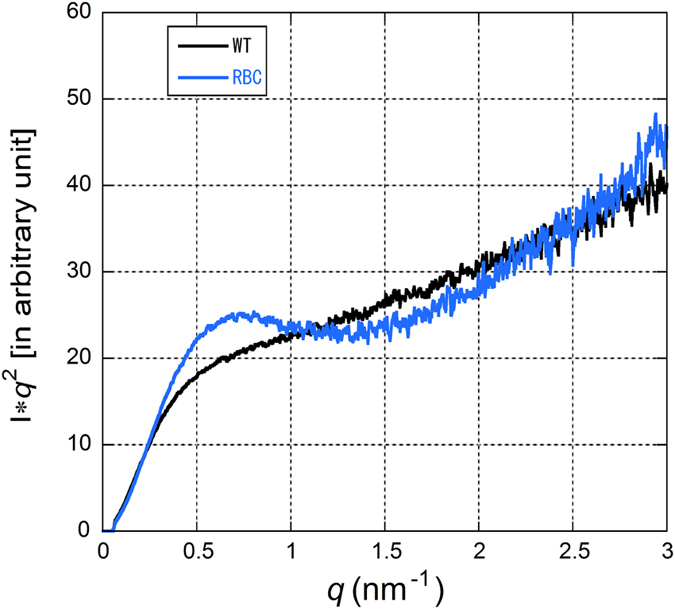
Kratky plots of recombinant α-syn (WT) and RBC α-syn (RBC) in the Tris buffer.

The value of R_g_ found in the static SAXS of the present study is similar to those reported previously^21^,^22^,^23^,^24^. As has been discussed, it is much larger than R_g_ values typically found in a globular protein with a similar molecular weight. For example, lysozyme, which was used for a calibration purpose in this study, gives an R_g_ value of 1.5–1.6 nm. This is taken as evidence that α-syn is partially unfolded in solution. However, there is a considerable variation in the reported R_g_ values which may be partially due to different buffer conditions. In the present study, we used two buffer conditions. One is the AA buffer with low ionic strength which was used by Bartels *et al.*^12^ in studies on human RBC α-syn. The other is a more widely used high ionic strength Tris buffer. The R_g_ value of the recombinant WT α-syn in the AA buffer was found to be much smaller than that in the Tris buffer (2.72 compared to 4.27 nm in the Tris buffer), even smaller than that of RBC α-syn in both buffers. These rather elusive results indicate that α-syn can take different conformations depending on its chemical environment. Figure 5 shows a comparison of Kratky plots of RBC and recombinant α-syn in the Tris buffer. It is clear that even RBC α-syn which has a smaller R_g_ is far from a compact, folded protein.

The SEC-SAXS result showed a smaller R_g_ value (3.59 nm) for recombinant WT α-syn than in a static measurement (4.27 nm). This may be due to removal of a fraction of recombinant α-syn that is forming oligomers in the high-ionic strength Tris buffer. The small absorption peak at an elution volume of about 10 ml (Fig. 4a) was consistently observed in separate experiments. Indeed, high ionic strength buffers are generally used in aggregation studies^24^,^25^,^26^. We found that addition of 150 mM NaCl to the AA buffer immediately caused aggregation. It might be possible to detect different populations with SEC-SAXS. Curtain *et al.* employed SEC-SAXS at low ionic strength and found two distinct conformations of α-syn, one with R_g_ smaller than 3 nm and the other larger than 4 nm^21^. It is likely that a similar variation of conformation also exists in the high ionic strength buffer. Giehm *et al.* studied fibrillation of α-syn in a high ionic strength buffer at 37 °C and separated monomer (with R_g_ of 4–5 nm) from oligomers^25^. For separation of different oligomers and conformations with SEC-SAXS, it is crucial to find an adequate flow rate because it is unclear how quickly the equilibrium among different populations is reached.

In conclusion, under two different conditions (high ionic strength with a Tris buffer and low ionic strength with an ammonium acetate buffer), no evidence was found for the presence of a tetramer. This means that a stable tetramer of α-syn cannot be found easily *ex vivo*. On the other hand, it was found that the buffer conditions strongly affect the radius of gyration of the protein. This suggests that the nature of α-syn, such as its folding structure, oligomerization and aggregation, is likely to considerably depend on the environmental condition. To investigate the pathogenesis of Parkinson’s disease, it is important to elucidate the nature of α-syn *in vivo*. For this reason, in the study of α-syn *in vitro*, it is necessary to pay sufficient attention to the environmental conditions.

## Methods

### Purification of recombinant wild-type α-synuclein

Human wild type (WT) α-syn was expressed in *E. coli* BL21(DE3) (Novagen)^27^. Cells were suspended in a purification buffer (50 mM Tris-HCl (pH 7.5), 1 mM EDTA, 0.1 mM dithiothreitol, and 0.1 mM phenylmethylsulfonyl fluoride), disrupted using sonication, and centrifuged (20,000 × g, 15 min). Streptomycin sulfate (final 2.5% (w/w)) was added to the supernatant to remove nucleic acids. After removal of nucleic acids by centrifugation, the supernatant was heated at 90 °C in a water bath for 15 min and then centrifuged. The supernatant was precipitated by addition of solid ammonium sulfate to 70% saturation, centrifuged, dialyzed overnight, and then applied onto a Resource-Q column (GE Healthcare) with 50 mM Tris-HCl buffer (pH 7.5) containing 0.1 mM dithiothreitol and 0.1 mM phenylmethylsulfonyl fluoride as a running buffer, and eluted with a linear gradient of 0–1 M NaCl. α-syn enriched fractions (as determined by SDS-PAGE/Coomassie Blue analysis) were then pooled and further purified by size exclusion chromatography using a Superdex 200 10/300 GL column (GE Healthcare) equilibrated with 50 mM Tris-HCl (pH 7.5), 150 mM NaCl. Pure fractions were combined and dialyzed against deionized water at 4 °C. The purity of the protein was confirmed to be higher than 95% by SDS-PAGE and MALDI mass spectroscopy (Fig. 1). Sample solution was then flash-frozen in liquid nitrogen and lyophilized.

### Purification of recombinant N-terminally acetylated α-synuclein

N-terminally acetylated α-syn was prepared as described^14^,^18^. The α-syn plasmid was co-expressed with the pNatB plasmid^28^. The two vectors were co-transformed into *E. coli* BL21 (DE3) using media containing both ampicillin and chloramphenicol. Subsequent purification method was the same as the above for WT α-syn.

### Blood sample collection

All experimental protocols for human samples were approved by the Ethical Review Board at Osaka University Graduate School of Medicine and were performed in accordance with the Ethical Guidelines for Clinical Research of the Ministry of Health, Labour and Welfare of Japan. Informed consent was previously obtained from all human subjects. Blood (200 ml or 400 ml) was collected from a healthy volunteer and white blood cells (WBCs) were depleted by Sepacell Integra CA (Asahi Kasei Medical) as the product manual. The flow diagram of purification of α-syn from the human RBCs is shown in Supplementary Figure S1.

### Lysis of human RBCs

The WBCs-depleted blood was centrifuged at 500 × g for 15 min at 4 °C and the serum was removed. The pelleted RBCs were washed three times by phosphate buffer solution (PBS). Then, RBCs were lysed by osmotic shock by addition of an equal volume of 5 mM phosphate buffer (pH 7.5) plus protease inhibitor mixture (Roche Applied Science). The solution was centrifuged at 20,000 × g for 15 min at 4 °C and the supernatant was collected and filtered through 0.22-μm filters.

### Hemoglobin depletion

RBC-soluble extracts were Hemoglobin (Hb) -depleted on nickel-based immobilized metal affinity chromatography as previously described^17^. Soluble extracts were loaded onto a 20-ml HisPrepTM FF 16/10 column (GE Healthcare).

### Purification of α-syn from Hb-depleted solution

Purification method was essentially the same as previously described^17^. First purification step was ion exchange chromatography (IEX). The Hb-depleted solution was injected onto a 5-ml HiTrap Q column (GE Healthcare) equilibrated with 50 mM Tris-HCl buffer, pH 7.5. α-Syn was eluted with a 0–1 M NaCl gradient in 50 mM Tris-HCl buffer (pH 7.5) (α-Syn eluted at ~0.30 M NaCl). α-syn enriched fractions (as determined by SDS-PAGE/Coomassie Blue analysis) were pooled for further purification by hydrophobic interaction chromatography (HIC). The pooled IEX fractions were injected onto a 5-ml HiTrap phenyl hydrophobic interaction column (GE Healthcare) equilibrated with 50 mM phosphate buffer (pH 7.0), 1 M (NH_4_)_2_SO_4_. α-syn was eluted with a 1–0 M (NH_4_)_2_SO_4_ gradient in 50 mM phosphate buffer (pH 7.0) (α-syn eluted at ~0.75 M (NH_4_)_2_SO_4_). α-syn enriched fractions (as determined by SDS-PAGE/Coomassie Blue analysis) were pooled for further purification by size exclusion chromatography (SEC). The pooled HIC fractions were injected onto a Superdex 200 10/300 GL column (GE Healthcare) equilibrated with 50 mM Tris-HCl (pH 7.5), 150 mM NaCl. Pure fractions were combined and dialyzed against appropriate solution at 4 °C. The purity of the solution was confirmed by SDS-PAGE and MALDI mass spectroscopy (Fig. 1a,b). It confirmed that the proteins were of high enough purity and that α-syn from human RBC (RBC) was likely to be N-terminally acetylated in terms of molecular weight. Sample solution was kept at 4 °C.

### Circular dichroism measurements

Far-UV Circular dichroism (CD) spectra were obtained with a Jasco 820 CD spectrophotometer as described previously^27^. Measurements were performed at 25 °C using a quartz cuvette with a 1 mm path length, and the results were expressed as mean residue ellipticity [θ].

### X-ray techniques

X-ray data were collected at a bending magnet beamline BL40B2 in SPring-8. The storage ring was run in a top-up mode. The X-ray beam was monochromatized with a double Si (111) monochromator and focused with a bent-cylindrical mirror. The X-ray energy was 12.4 keV. The energy resolution was about 1 × 10^−4^. The sample-to-detector distance was 1650 mm. The flux was about 5 × 10^10^ photons/sec. The beam size was about 0.2 mm in diameter. The q-spacing was calibrated with diffraction from silver behenate (first peak at d = 5.84 nm). All experiments were made at 25 °C.

For static measurements, α-syn was dissolved in either the Tris buffer (50 mM Tris-HCl (pH 7.5), 150 mM NaCl) or the AA buffer (10 mM ammonium acetate, pH 7.4). The sample solution was contained in a cell with 0.02 mm-thick quartz windows. The volume of the cell was about 30 μl. The exposure time was 120 s. An image plate X-ray detector (RAXIS-VII, RIGAKU, Japan) with a pixel size of 100 μm × 100 μm was used. Multiple measurements were made at two to four different protein concentrations.

For SAXS coupled with a size-exclusion column (SEC-SAXS), the protein solution was loaded onto a size exclusion column (Superdex 200 10/300 GL) and eluted in the Tris buffer with a pump for a SAXS measurement. The X-ray detector was PILATUS-100K (DECTRIS, Switzerland). About 8 mg of α-syn was loaded to the column and the eluted solution was passed through a polyimide microtube (LB grade, KN01-LB20, Protein Wave co., Nara, Japan) with a diameter of 2 mm and a wall thickness 0.1 mm for X-ray measurement at a flow rate of 0.2 ml/min. The UV absorption at 280 nm was continuously monitored between the column and the polyimide microtube. An X-ray scattering measurement at a frame rate of 10.003 sec (the exposure time 10 sec) was started when the protein concentration began to increase. The scattering data acquired when the protein concentration was more than half of the peak concentration were later summed for analysis.

### Data analysis

Scattering data were averaged circularly and subjected to analysis by a software package PRIMUS^29^. The radius of gyration (R_g_) and the forward scattering intensity (I(0)) were obtained from a Guinier plot. With each protein sample, measurements were made at more than three different concentrations for each batch of preparation. R_g_ and I(0)/c (c is concentration) were plotted against the concentration and fitted with a straight line to obtain values at zero concentration (Fig. 4). Mean values of R_g_ and I(0)/c were obtained by averaging the zero concentration values from different preparations.

The forward scattering intensity of α-syn was evaluated by comparison with that from hen egg lysozyme (six-times crystallized, Seikagaku-Kogyo, Japan), which has a molecular weight (14.3 kDa) similar to α-syn. Measurements were made at more than three concentrations of lysozyme and I(0)/c at zero concentration was obtained.

## Additional Information

**How to cite this article**: Araki, K. *et al.* A small-angle X-ray scattering study of alpha-synuclein from human red blood cells. *Sci. Rep.* **6**, 30473; doi: 10.1038/srep30473 (2016).

## Supplementary Material

Supplementary Information

## Figures and Tables

**Table 1:** Summary of results on R_g_ measurements (in nm).

		static SAXS			SEC-SAXS	
		WT	NAc	RBC	WT	NAc
**Tris buffer**	mean	4.27	4.04	3.31	3.59	3.59
	SD	0.37	0.22	0.3	0.14	0.28
	n	7	4	4	5	2
**AA buffer**	mean	2.72	3.09	3.33		
	SD	0.44	0.18	0.10		
	n	3	2	2		

WT, NAc and RBC represent wild type α-syn, N-terminally acetylated α-syn and α-syn purified from human RBCs, respectively.

## References

[b1] Spillantini M. G., Crowther R. A., Jakes R., Hasegawa M. & Goedert M. α-Synuclein in filamentous inclusions of Lewy bodies from Parkinson disease and dementia with Lewy bodies. Proc. Natl. Acad. Sci. 95, 6469–6473 (1998). 9600990 10.1073/pnas.95.11.6469 PMC27806

[b2] Baba M. *et al.* Aggregation of α-synuclein in Lewy bodies of sporadic Parkinson disease and dementia with Lewy bodies. Am. J. Pathol. 152, 879–884 (1998). 9546347 PMC1858234

[b3] Araki K. *et al.* Synchrotron FTIR micro-spectroscopy for structural analysis of Lewy bodies in the brain of Parkinson’s disease patients. Sci Rep. 5, 17625 (2015). 26621077 10.1038/srep17625 PMC4664933

[b4] Tuttel M. D. *et al.* Solid-state NMR structure of a pathogenic fibril of full-length human α-synuclein. Nat Struct Mol Biol. 23, 409–415 (2016). 27018801 10.1038/nsmb.3194 PMC5034296

[b5] Maroteaux L., Campanelli J. T. & Scheller R. H. Synuclein – a neuron-specific protein localized to the nucleus and presynaptic nerve terminal. J. Neurosci. 8, 2804–2815 (1988). 3411354 10.1523/JNEUROSCI.08-08-02804.1988 PMC6569395

[b6] Iwai A. *et al.* The precursor protein of non-A beta component of Alzheimer’s disease amyloid is a presynaptic protein of the central nervous system. Neuron 14, 467–475 (1995). 7857654 10.1016/0896-6273(95)90302-x

[b7] Bottner M. *et al.* Expression pattern and localization of alpha-synuclein in the human enteric nervous system. Neurobiol. Dis. 48, 474–480 (2012). 22850485 10.1016/j.nbd.2012.07.018

[b8] Barbour R. *et al.* Red blood cells are the major source of alpha-synuclein in blood. Neurodegener. Dis. 5, 55–59 (2008). 18182779 10.1159/000112832

[b9] Conway K. A., Harper J. D. & Lansbury P. T. Accelerated *in vitro* fibril formation by a mutant alpha-synuclein linked to early-onset Parkinson disease. Nat. Med. 4, 1318–1320 (1998). 9809558 10.1038/3311

[b10] Giasson B. I., Uryu K., Trojanowski J. Q. & Lee V. M. Mutant and wild type human alpha-synucleins assemble into elongated filaments with distinct morphologies *in vitro*. J. Biol. Chem. 274, 7619–7622 (1999). 10075647 10.1074/jbc.274.12.7619

[b11] Manning-Bog A. B. *et al.* The herbicide paraquat causes up-regulation and aggregation of alpha-synuclein in mice: paraquat and alpha-synuclein. J. Biol. Chem. 277, 1641–1644 (2002). 11707429 10.1074/jbc.C100560200

[b12] Bartels T., Choi J. G. & Selkoe D. J. α-Synuclein occurs physiologically as a helically folded tetramer that resists aggregation. Nature 477, 107–110 (2011). 21841800 10.1038/nature10324 PMC3166366

[b13] Wang W. *et al.* A soluble α-synuclein construct forms a dynamic tetramer. Proc. Natl. Acad. Sci. 108, 17797–17802 (2011). 22006323 10.1073/pnas.1113260108 PMC3203798

[b14] Trexler A. J. & Rhoades E. N-Terminal acetylation is critical for forming α-helical oligomer of α-synuclein. Protein. Sci. 21, 601–605 (2012). 22407793 10.1002/pro.2056 PMC3403458

[b15] Marotta N. P. *et al.* O-GlcNAc modification blocks the aggregation and toxicity of the protein α-synuclein associated with Parkinson’s disease. Nat Chem. 11, 913–920 (2015). 26492012 10.1038/nchem.2361 PMC4618406

[b16] Lee S. J. *et al.* Probing Conformational Change of Intrinsically Disordered α-Synuclein to Helical Structures by Distinctive Regional Interactions with Lipid Membranes. Anal. Chem. 86, 1909–1916 (2014). 24383916 10.1021/ac404132g

[b17] Fauvet B. *et al.* Alpha-synuclein in the central nervous system and from erythrocytes, mammalian cells and *E. coli* exists predominantly as a disordered monomer. J. Biol. Chem. 287, 15345–15364 (2012). 22315227 10.1074/jbc.M111.318949 PMC3346117

[b18] Fauvet B. *et al.* Characterization of semisynthetic and naturally Nα-acetylated α-synuclein *in vitro* and in intact cells: implications for aggregation and cellular properties of α-synuclein. J. Biol. Chem. 287, 28243–28262 (2012). 22718772 10.1074/jbc.M112.383711 PMC3436566

[b19] Burré J. *et al.* Properties of native brain α-synuclein. Nature 498, E4–E6 (2013). 23765500 10.1038/nature12125 PMC4255827

[b20] Theillet F. X. *et al.* Structural disorder of monomeric α-synuclein persists in mammalian cells, Nature 530, 45–50 (2016). 26808899 10.1038/nature16531

[b21] Curtain C. C. *et al.* Alpha-synuclein oligomers and fibrils originate in two distinct conformer pools: a small angle X-ray sscattering and ensemble optimisation modelling study. Mol. Biosys. 11, 190–196 (2015). 10.1039/c4mb00356j 25352253

[b22] Rekas A. *et al.* The structure of dopamine induced alpha-synuclein oligomers. Eur. Biophys. J. 39 1407–1419 (2010). 20309679 10.1007/s00249-010-0595-x

[b23] Tashiro M. *et al.*, Characterization of fibrillation process of α-synuclein at the initial stage. Biochem. Biophys. Res. Comm. 369, 910–914 (2008). 10.1016/j.bbrc.2008.02.127 18329380

[b24] Uversky V. N., Li J. & Fink A. L. Evidence for a partially folded intermediate in alpha-synuclein fibril formation. J Biol Chem. 276, 10737–10744 (2001). 11152691 10.1074/jbc.M010907200

[b25] Giehm L., Svergun D. I., Otzen D. E. & Vestergaard B. Low-resolution strcture of a vesicle disrupting α-synuclein oligomer that accumulated during fibrillation. Proc. Nat. Acad. Sci. 108, 3246–3251 (2011). 21300904 10.1073/pnas.1013225108 PMC3044375

[b26] Nielsen S. B. *et al.* Wildtype and A30P mutant alpha-synuclein form different fibril structures. PLoS One 8, e67713 (2013). 23861789 10.1371/journal.pone.0067713 PMC3701545

[b27] Yagi H., Kusaka E., Hongo K., Mizobata T. & Kawata Y. Amyloid fibril formation of alpha-synuclein is accelerated by preformed amyloid seeds of other proteins: implications for the mechanism of transmissible conformational diseases. J. Biol. Chem. 280, 38609–38616 (2005). 16162499 10.1074/jbc.M508623200

[b28] Johnson M., Coulton A. T., Geeves M. A. & Mulvihill D. P. Targeted amino-terminal acetylation of recombinant proteins in *E. coli*. PLoS One 5, e15801 (2010). 21203426 10.1371/journal.pone.0015801 PMC3009751

[b29] Konarev P. V. *et al.* PRIMUS: a Windows PC-based system for small-angle scattering data analysis. J. Appl. Cryst. 36, 1277–1282 (2003).

