# Supplementary material for: A small-angle X-ray scattering study of alpha-synuclein from human red blood cells
Source: Sci Rep. 2016 Jul 29;6:30473. doi: 10.1038/srep30473 (PMC4965831; doi:10.1038/srep30473)
Supplement: Supplementary Information [file srep30473-s1.doc]

**Supplementary Information for**

**A small-angle X-ray scattering study of alpha-synuclein from human red blood cells**

Katsuya Araki ^1^, Naoto Yagi ^2^*, Rie Nakatani ^1^, Hiroshi Sekiguchi ^2^, Masatomo So ^3^, Hisashi Yagi ^4^, Noboru Ohta ^2^, Yoshitaka Nagai ^5^, Yuji Goto ^3^, and Hideki Mochizuki ^1^*

^1^ Department of Neurology, Osaka University Graduate School of Medicine, 2-2 Yamadaoka, Suita, Osaka 565-0871, Japan

^2^Japan Synchrotron Radiation Research Institute (JASRI), SPring-8, 1-1-1 Kouto, Sayo, Sayo, Hyogo 679-5198, Japan

^3^ Institute for Protein Research, Osaka University, 3-2 Yamadaoka, Suita, Osaka 565-0871, Japan

^4^ Center for Research on Green Sustainable Chemistry, Tottori University, 4-101 Koyamacho-minami, Tottori, Tottori 680-8550, Japan

^5^ Department of Neurotherapeutics, Osaka University Gradf Medicineuate School o, 2-2 Yamadaoka, Suita, Osaka 565-0871, Japan

**Supplementary Figure S1**

Left figure shows a flow diagram of the purification of α-syn from human RBCs (left). The right figures are SDS-PAGE / Coomassie (or silver) staining after Ion exchange chromatography (IEX), Hydrophobic interaction chromatography (HIC) and Size exclusion chromatography (SEC).

**
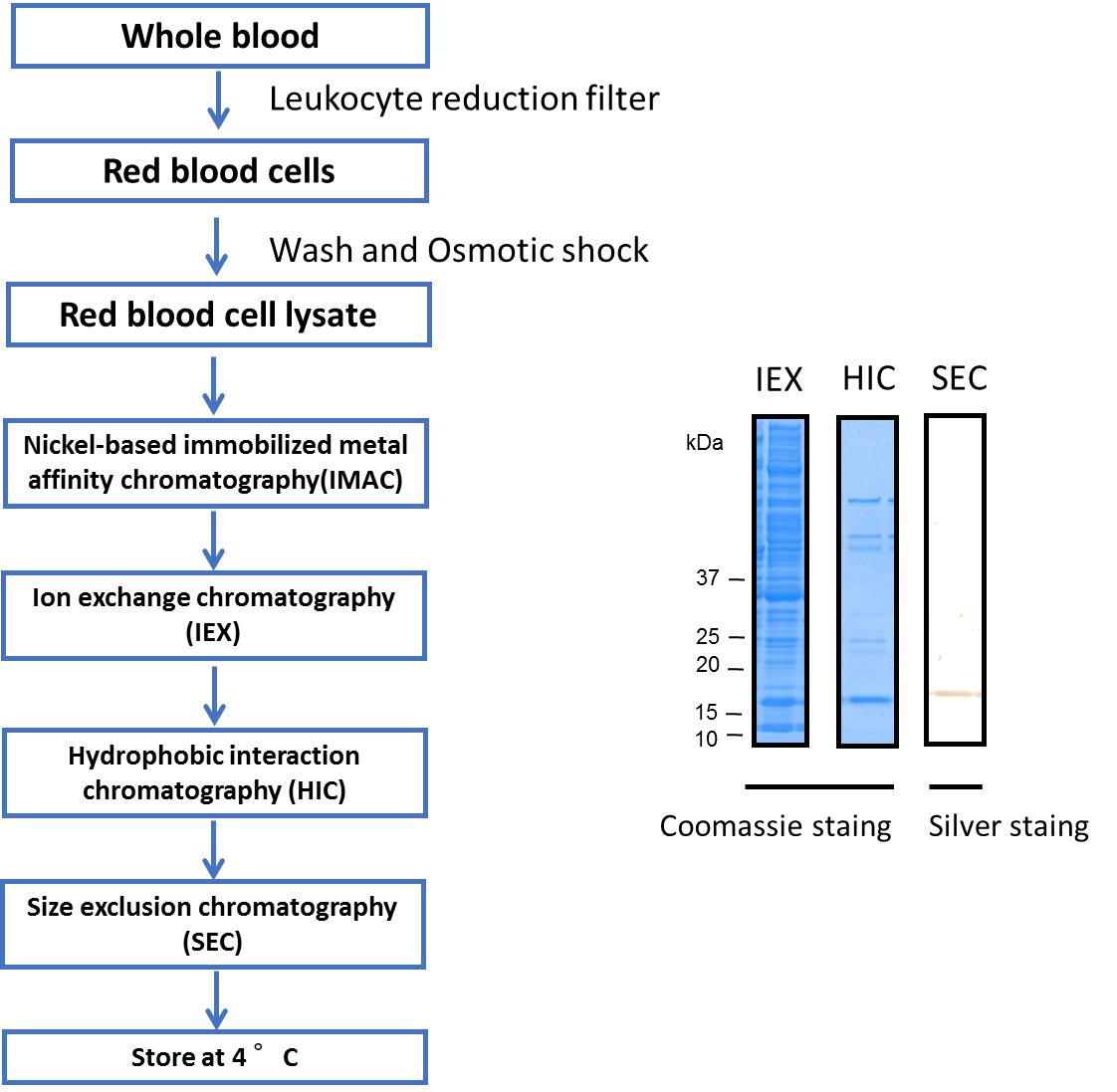
**
